# The Critical Role of Pharmacists in the Clinical Delivery of Pharmacogenetics in the U.S

**DOI:** 10.3390/pharmacy11050144

**Published:** 2023-09-10

**Authors:** Susanne B. Haga

**Affiliations:** Division of General Internal Medicine, Department of Medicine, School of Medicine, Duke University, 101 Science Drive, Durham, NC 27708, USA; sbhaga@duke.edu

**Keywords:** pharmacogenetic testing, pharmacist, barriers, education

## Abstract

Since the rebirth of pharmacogenomics (PGx) in the 1990s and 2000s, with new discoveries of genetic variation underlying adverse drug response and new analytical technologies such as sequencing and microarrays, there has been much interest in the clinical application of PGx testing. The early involvement of pharmacists in clinical studies and the establishment of organizations to support the dissemination of information about PGx variants have naturally resulted in leaders in clinical implementation. This paper presents an overview of the evolving role of pharmacists, and discusses potential challenges and future paths, primarily focused in the U.S. Pharmacists have positioned themselves as leaders in clinical PGx testing, and will prepare the next generation to utilize PGx testing in their scope of practice.

## 1. Introduction

Although pharmacogenetics (PGx) has been around a long time, the field of study known as PGx was actually established during the 1950s, with several reports on the role of genetic factors in the risk of adverse events and response to medications [1,2,3]. Decades of research on patient subsets with adverse events and poor responses solidified the key role of enzymes, transporters, and other key proteins in drug metabolism and transport. With the onset of new sequencing technologies and the completion of the Human Genome Project, the field of pharmacogenetics benefitted tremendously, with new research able to analyze many genes in large patient populations to better define the scope of key variants and their contribution to medication response, thereby transitioning to the field of ‘pharmacogenomics’. Soon thereafter, clinical tests were developed, and the era of clinical PGx testing and clinical implementation began.

The availability of PGx testing, however, has not been sufficient to catapult it into routine use. Globally, studies on the clinical implementation of PGx testing have revealed a number of challenges including limited provider knowledge, evidence, reimbursement and clinical decision support [4,5,6,7,8,9,10,11]. Many health system infrastructures do not include operational features to link PGx test results to prescriptions in order to enable prescribers to identify the potential risk of adverse events or non-response and thus make an informed treatment decision based on a patient’s test results (along with other clinical factors). In addition, there has not been a clear path for the clinical delivery of PGx testing, as it straddles the provider (prescriber) and pharmacist communities. However, pharmacists have played a key role in the advent of pharmacogenetics and later pharmacogenomics, and have defined a path forward for clinical implementation. Currently, pharmacists continue to play a key role in establishing, launching, evaluating, consulting, and leading clinical delivery of PGx testing in a variety of clinical settings.

Since 2015, the American Society of Health-System Pharmacists (ASHP) has recognized the role of pharmacists in the delivery of PGx testing [12]: “Because of their distinct knowledge, skills, and abilities, pharmacists are uniquely positioned to lead interprofessional efforts to develop processes for ordering pharmacogenomic tests and for reporting and interpreting test results” [13]. This paper provides a brief overview of pharmacists’ roles to date, the challenges encountered, and a vision for the future.

## 2. Pharmacist Roles and Implementation Studies

Given the early involvement and leadership of pharmacists in PGx research through the development of research groups such as the Pharmacogenomics Global Research Network (PGRN) in the late 1990s, the continuation of pharmacists’ leadership and participation in the clinical use of PGx testing has been anticipated. Several PGx pharmacist researchers have transitioned into leading clinical roles, serving as leaders of PGx clinical programs at multiple institutions including the University of Florida and St Jude Children’s Hospital. In the U.S., the Clinical Pharmacogenetics Implementation Consortium (CPIC) is an organization that was established by leading pharmacist researchers in PGx; about half of participating sites are led by pharmacists [14]. Institutional leadership roles may involve a variety of responsibilities, including working with the clinical laboratories to set up a PGx test, negotiating with health system executives to create a PGx testing program, serving as the lead educator of providers, and developing new training programs for pharmacist residents in PGx.

Various other roles for pharmacists have been described in the literature [15], including initiating and/or ordering PGx tests, providing counseling to patients, interpreting test results, making treatment recommendations based on the results, consulting with prescribers, and serving as an expert resource to both patients and providers (Table 1). These various roles pivot depending on the type of clinical setting they work in, which in turn impacts their relationship with patients and/or prescribers and affects when testing is ordered along the spectrum of care.

**Figure 1:**
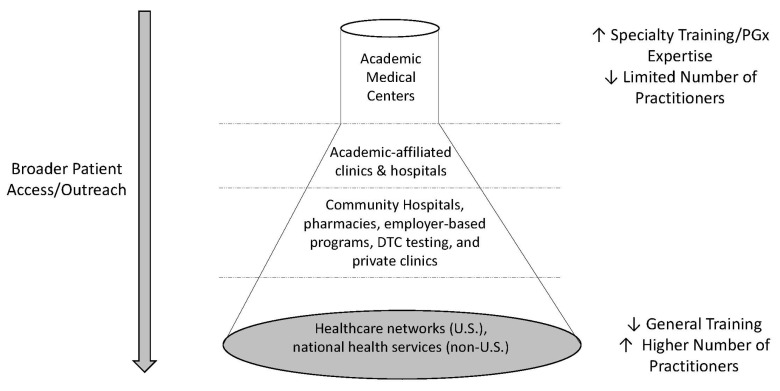
The introduction of PGx testing originated in academic medical centers and has dispersed to other settings, with greater patient outreach and access.

With respect to their roles in the clinical delivery of PGx testing, pharmacists have been involved or led the delivery of PGx testing in three major settings (Figure 1): (1) health systems or hospital-based pharmacist services [16,17,18,19,20,21,22,23]; (2) outpatient clinic-based consultation services or clinics [24,25,26,27,28,29,30]; and (3) community pharmacies [31,32,33,34,35,36,37,38,39]. Many PGx programs were initiated and reside in departments and divisions of academic medical centers where many leading pharmacist researchers are based (Figure 1). These centers are most likely to have the expertise and training programs required to establish a PGx testing program and support the required infrastructure. Subsequently, PGx testing may spread to academic-affiliated clinics before moving outside of academic medicine to community-based settings where larger patient populations will have access to testing.

In addition, pharmacists have been involved with the delivery of PGx testing across a range of clinical specialties, namely cardiology, psychiatry, pain management, and polypharmacy (see the review by Hayashi et al., 2022) [40]. Thus, as pharmacists may work in multiple clinical settings, there is no single role for the pharmacist or mode of delivery of PGx testing.

With respect to when PGx testing is ordered during the journey of care, testing can be ordered in advance of needing medication (preemptive testing), at the time of treatment (the point of care), or post-treatment, in order to understand the causes of an adverse event or non-response [41]; pharmacists have mostly been involved in the delivery of PGx testing at the time of treatment. Many studies to date have been conducted to evaluate the role of pharmacists in the delivery of PGx testing across a range of roles and clinical settings. 

*Health system- or hospital-based settings.* In several hospital or health system settings, pharmacists have served or led consultation services, or established a PGx “clinic” to advise health providers and/or offer counseling services to patients about PGx testing. For example, a health system in Chicago (Illinois, USA) established a multi-disciplinary PGx clinic that included a medical geneticist, genetic counselors, and a PGx-trained pharmacist, based in the health system’s Center for Medical Genetics [42]. Pharmacists in this service directly met with patients. In contrast, a pharmacist-managed clinical PGx service was created at St Jude Children’s Research Hospital (Tennessee, USA), which worked closely with the testing laboratory and provided interpretation of and recommendations for each result, and communicated to the treating providers. [19]. In addition to hospital- and health system-based services, programs have been launched on the national level. Bain et al. established a pharmacist-led service for community-based Program of All-Inclusive Care for the Elderly (PACE) centers around the country to serve as a resource for prescribers [43]. For PACE patients, prescribers would order the PGx test, but the pharmacists provided education and interpretation of results. In-hospital, multi-disciplinary PGx consultations have also been established in several locations, with pharmacist members assisting providers by implementing PGx results into treatment decisions [18]. Some programs are focused on one clinical specialty (e.g., cardiology), while others are not linked to any specialty or medication class.

*Ambulatory or Outpatient Settings.* In some outpatient locations, pharmacists serve as members of the clinical care team [44]. Within this smaller clinical setting, pharmacists may provide medication counseling services directly to patients, and/or advise prescribers on complex cases. Primary care is one clinical setting that has been evaluated in multiple publications [45]. Given the high rate of prescriptions written by primary care physicians, it is not surprising to find this to be a common setting for evaluating the delivery of PGx testing involving pharmacists. In one example of a PGx service available to multiple primary care clinics affiliated with a community hospital, pharmacists were responsible for the review and communication of PGx test results to the ordering provider [25]. Studies have reported the integration and acceptance of pharmacists in the clinic setting, including adding PGx testing to existing services [24,46]. 

*Community Pharmacy Settings.* Several studies have explored the role of delivering PGx testing in the community pharmacy setting. In this setting, pharmacists may introduce PGx testing to patients who present with prescriptions for medications with known PGx impact. In contrast to physicians and other health providers, pharmacists may be more accessible to patients [47] and are reported to be a trusted resource of health information [48,49]. Furthermore, as a result of stronger relationships with patients and knowledge of their medication history, pharmacists may be better able to identify patients who may benefit from PGx testing. Studies have reported high satisfaction from customers/patients undergoing PGx testing in the community pharmacy setting [46,50].

In both the outpatient setting and community pharmacies, the integration of PGx testing into medical therapy management (MTM) services has been promoted [51]. Several groups have evaluated the feasibility of adding PGx testing to MTM, and generally have reported favorable outcomes [24,52,53,54]. Depending on the setting, the delivery of MTM plus PGx testing may be challenging to implement [55], but would enable more careful reviews of patients’ medication history, and may present the opportunity to discuss PGx testing in detail with the patient, as well as consider the potential risks posed by PGx variants to current and future medications. Pharmacist-led medication reconciliation with PGx testing has also been demonstrated to be an effective approach to identifying medication discrepancies and adjustments based on test results [56]. 

In a slight variation of the community pharmacist role, with the pending availability of direct-to-consumer (DTC) PGx testing and patient self-ordered testing (where the results are directly returned to the consumer/patient), community pharmacists may again be called upon to advise patients about the significance of their results to their medications, and/or may serve as a resource for physicians to manage these results with respect to medications [57]. DTC PGx testing kits may be sold and promoted in pharmacies; therefore, the pharmacist is likely to be a logical and convenient resource for consumers/patients. 

*Non-U.S.-Based Settings.* While the prior examples are U.S.-based, it should be recognized that there have been many efforts around the world to evaluate the role of pharmacists in the clinical delivery of PGx testing. Despite differences in health care systems, many non-U.S. PGx implementation studies involving pharmacists are similar with respect to their role, relationships with prescribers, and patient experiences. For example, several Dutch studies have evaluated the use of pharmacist-initiated PGx testing for healthy patients [58,59], as well as point-of-care testing [60]. One Swiss study evaluated the delivery of PGx testing via a community pharmacy [61], and another study developed an interprofessional pharmacist-led PGx service [62]. In Norway, a study assessed the feasibility of incorporating PGx testing into pharmacist-led medication reviews of hospitalized patients [63]. In Canada, studies have evaluated the feasibility of a PGx service clinic with pharmacists [64] and evaluating PGx testing in a community pharmacy setting [65]. A more complex system involving both hospital and community pharmacists delivering PGx testing was studied in a Spain [66]. Thus, these few examples illustrate the range of services offered by pharmacists in the delivery of PGx testing around the globe.

One unique feature, however, of PGx implementation studies outside of the U.S. is the establishment of national programs. This is obviously enabled by a centralized or national health care system present in many countries. In the UK, PGx services have been delivered through local community pharmacists services [36,67], but plans are underway to develop a national program through the National Health Service (NHS) [68,69]. Specifically, the Royal College of Physicians issued a report in 2022, recommending the implementation of PGx testing nationwide to enable PGx-based prescribing [70]. NHS Wales currently offers some PGx tests for cancer treatments to all patients [71]. Several European countries are participating in a program called the PREemptive Pharmacogenomic testing for prevention of Adverse drug REactions [PREPARE], operated by the Ubiquitous Pharmacogenomics (U-PGx) Consortium [72]. The most recent findings, involving multiple sites including 28 community pharmacies in seven European countries (Austria, Greece, Italy, the Netherlands, Slovenia, Spain, and the UK), showed a significantly reduced incidence of adverse outcomes, from 27% in the control group to 21% in the PGx-guided group [73].

## 3. Pharmacist Barriers

**Figure 2:**
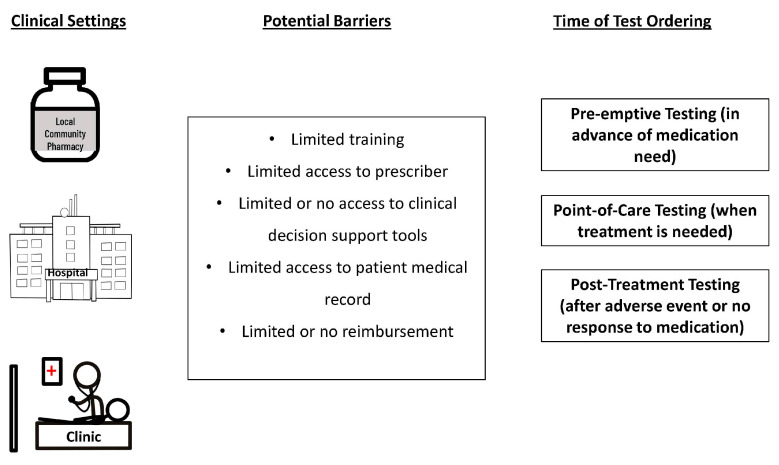
Illustration of the multiple options with respect to the clinical setting and time of testing along the spectrum of care, and potential barriers for pharmacists in the delivery of PGx testing. Note that not all combinations are possible for every setting, given unique circumstances and infrastructures. Likewise, not all listed barriers are applicable to each setting or type of testing.

Despite strong recommendations that pharmacists serve as leaders in the clinical delivery of PGx testing, some barriers have been identified [74,75,76,77]. In particular, limited knowledge about PGx testing is an oft-cited barrier to pharmacists’ implementation of PGx testing [14,78,79,80,81,82,83,84,85]. However, the pharmacist community has been very proactive in increasing training opportunities and curriculum content, as discussed in the next section. However, there are several other individual and setting-related factors that have posed challenges to pharmacists’ delivery of PGx testing. Barriers will vary depending on the clinical setting and the time when PGx testing is ordered (Figure 2).

*Time.* Logistically, time has been raised as a potential concern for community pharmacists, with some pharmacists feeling that PGx testing is too time-consuming to incorporate into current practice [86]. In the past decade, pharmacists’ scope of services has expanded beyond medication dispensing to include vaccinations, diabetes and hypertension management, smoking cessation, obesity and chronic disease screening, and even prescribing [87,88]. In particular, the additional patient time required to offer PGx testing and determining how best to integrate PGx testing into their workflow are major concerns that have been raised by pharmacists [89]. Liko et al. [27] reported that pharmacists met with patients for two 1 h sessions, one before testing and one after testing. Bright et al. [35] assessed the time taken for a PGx consultation service offered by community pharmacists, and reported that an average of 10 min was spent per patient. In contrast, testing through a multi-disciplinary PGx clinic may entail an hour-long office visit for pre- and post-testing discussions [42]. However, this additional time could be significantly reduced through the incorporation of support services from a pharmacy technician [35]. The development of point-of-care testing (POCT) may eliminate the need for a second visit with the pharmacist, and thus substantially reduce overall time requirements [35,60].

*Pharmacist–Prescriber Relationships.* In addition to patient time, the time required to consult with the prescriber/provider before and after testing also needs to be considered. In the U.S., clinical tests are not typically ordered by pharmacists, and their authority is defined by state regulations. Collaborative practice agreements or partnerships with physician groups may enable tests to be ordered by pharmacists for specific conditions. During the pandemic, the U.S. Department of Health and Human Services decreed that pharmacists had the authority to order COVID-19 diagnostic testing under the federal Public Readiness and Emergency Preparedness Act [90]. Thus, the initiation of the PGx testing process may warrant authorization from the prescriber if the pharmacist is not an authorized provider. However, the reported limited understanding of physicians [7,91,92,93,94,95,96,97,98] and differences in the perceived benefits of PGx testing between physicians and pharmacists pose challenges to pharmacist-initiated PGx testing [89]. In contrast to a team-based clinical setting, the community pharmacist may or may not have a relationship with the prescribing provider, but may have a very close relationship with the customer/patient, as noted earlier. Some community pharmacies operate in stores that also offer health services, presenting an opportunity to collaborate with providers at the same location or within the same health network. 

The pharmacist–prescriber relationship is also central to the application of PGx results to treatment decisions, but has been reported as a challenge. While pharmacists and physicians have established close working relationships for other clinical services and pharmacists’ contributions are valued by physicians [99], their respective roles in the delivery of PGx testing in particular are somewhat unclear [75,100]. Studies have reported a range of acceptance rates for treatment recommendations based on a test that is initiated and interpreted by a pharmacist and communicated to the prescribing provider [19,28,32,43,52,59,101,102,103], likely impacted by the type of relationship between prescriber and pharmacist and the severity of medication risk. In one program, a hospital pharmacist and prescriber jointly reviewed patients’ PGx test results with their full medical histories to determine if medication changes were needed [104]. The lack of provider acceptance may be due to lack of prescriber knowledge about PGx testing [7,84,91,92,93,94,95,96,97,98,105] and the unclear roles involved in the delivery of PGx testing. To address this challenge, one pharmacist-led consultation service included physician education as a part of their service to help physicians identify patients likely to benefit from PGx testing and promote therapeutic decision-making based on PGx test results [43]. 

More recently, following the pandemic and pharmacists’ expanded authority regarding testing services [106], it is possible that this experience will advance a greater role for pharmacists with respect to clinical testing in general, and more specifically for PGx testing. However, while the continued expansion of pharmacy services to include testing may yield economic benefits for pharmacies and increase access to various services for patients [107,108], an expanded role beyond the traditional roles of dispensation and patient counseling (via community-based pharmacists) [109] may not be wholly supported by physicians, and factors such as pharmacists’ clinical expertise and control influence physicians’ attitudes [110,111]. However, in the community setting (or a centralized service serving multiple sites), pharmacists are not likely to have access to a patient’s full medical record or medication history, thereby limiting their ability to interpret the clinical significance of the PGx test results [43]. Community pharmacists may also not have a close working relationship with a prescribing physician, which may further impede implementation of PGx testing [89]. In contrast, in outpatient clinic settings, pharmacists may serve as members of clinical teams, and have access to both the patient’s medical record and health provider.

In contrast to the active efforts of the pharmacist community (described in the next section), there has been little effort to increase PGx content in curricula, or to develop physician training and education programs. Another reason for the uneven acceptance rate of PGx recommendations by prescribers is that physicians may perceive fewer favorable benefits of PGx testing than pharmacists [60,112]. Due to the need for physician authorization of PGx test orders initiated by a pharmacist, uneven understanding and differences in perceived benefits pose challenges [89]. 

In addition to the relationship with prescribers, another group of providers that has been discussed regarding the delivery of PGx testing and pharmacists are genetic counselors. In particular, genetic counselors’ expertise in testing, patient communication and test interpretation could fulfill a gap in pharmacists’ knowledge of genetics and genetic testing [113,114,115]. Likewise, pharmacists’ deep understanding of medication pathways and adverse events fill a void in genetic counselors’ knowledge. Thus, a team-based delivery of PGx testing between pharmacists, genetic counselors, and other providers may yield a more comprehensive service than that of any group individually [42]. However, the limited number of genetic counselors, limited access (primarily based in academic medical centers and in urban areas), and reimbursement issues would limit the widespread establishment of these partnerships, particularly in community clinical settings. 

*Clinical Decision Support (CDS).* Having the appropriate clinical decision support tools will be critical to pharmacists’ ability to integrate PGx test results into their reviews and fulfillment of new prescriptions. In particular, pharmacists have recognized the need for software to send out alerts about testing recommendations for new medications impacted by PGx and potential risks for new medications [116], as well as the need for additional education to appropriately use digital resources to appropriately apply test results [89]. CDS systems are needed in all types of clinical settings to facilitate the integration and use of PGx test results. A pharmacist-directed decision support system has been demonstrated to be feasible and useful at two hospice sites [117]. In the community-based Program of All-Inclusive Care for the Elderly (PACE), a clinical decision support system enabled identification of drug–gene and drug–drug–gene interactions [118]. More complex treatment scenarios such as polypharmacy can greatly benefit from clinical decision support software [101] and the identification of patients at higher risk [52]. Preemptive testing in particular may benefit from clinical decision support in the identification of newly prescribed medications that may be impacted by a patient’s PGx status [119].

Efforts have been underway to address a similar need for physicians as well [120], and many implementation studies include some type of active clinical decision support [103,119,121,122,123,124]. For the U-PGx project, a multi-national clinical decision support was developed and tested in 15 sites across seven EU countries [125]. Personalized PGx test reports could be generated in 20 min in multiple languages, though institutional-specific factors presented some challenges [125]. Some clinical decision supports with alerts have been designed to be less interruptive, potentially yielding greater acceptance [126].

Despite the benefits associated with CDS, there are substantial challenges to the development and acceptance/utilization of these tools [127,128,129,130,131]. Clinical decision support developers face issues such as designing a friendly user interface, extracting free text in the medical record, prioritizing clinical recommendations, maintenance, and drafting appropriate language for alerts, as well as providing additional informational sources for providers and patients. For CDS for PGx testing, lack of standardization and physician knowledge may impact acceptance/follow-up of CDS alerts [132]. Usability testing is essential to optimizing utilization [133]. 

*Reimbursement.* One additional barrier that has been noted in both US and non-US settings is the issue of reimbursement for non-dispensing services, such as pharmacist PGx-related services [76,105,134,135]. While there are specific services that pharmacists can directly receive reimbursement for, such as MTM, and/or they can bill through a physician’s office, the reimbursement structure for PGx-related services specifically is unclear. If PGx-related services are embedded in other services such as MTM, reimbursement could be obtained for MTM, but would not cover the additional time required to include PGx counseling, interpretation of results, and therapeutic recommendations. In addition, there are inconsistent coverage policies for the actual testing, and the cost would have to be covered by the patient out-of-pocket. The additional time of pharmacists can be quite significant, and unfeasible without some reimbursement; therefore, this issue needs to be addressed in order for pharmacists to be able to offer these services [135]. Another option is to establish a fee-for service practice, as reported by Schuh and Crosby [136], in an office-based clinical setting. In addition, some work has even demonstrated cost avoidance as a positive outcome of a pharmacist-led PGx services [137].

## 4. Enhancing PGx Content in the Pharmacy Curricula and Continued Learning Opportunities

As the roles of pharmacists became more defined, with a general belief that this group will play a key role in the delivery of PGx testing as well as continuing to advance research, there have been several calls for enhanced training and workforce preparedness. Dating back to 2002, the Academic Affairs committee of the American Association of Colleges of Pharmacy (AACP) was tasked to consider the educational needs of pharmacists to prepare for the use of pharmacogenetics [138]. Around this time, the AACP had been involved in efforts by the National Coalition for Health Professional Education in Genetics to develop competencies for health providers [139]. Thus, the pharmacist community was working to ensure that future practitioners would be equipped with the knowledge and skills to utilize these new tools in practice. Fast forward to 2015, when the American Society of Health-System Pharmacists issued a statement regarding pharmacists’ role in clinical PGx [12]. Shortly thereafter, in 2016, pharmacogenomics was added as a required element to the Didactic Doctor of Pharmacy Curriculum [140]. Pharmacogenomics competencies in pharmacy practice were published in 2017 [141] and updated in 2021 [142]. In 2021, Rahma et al. developed a ‘health literacy skills framework’ for pharmacists [143].

These efforts have led to greater inclusion of PGx content in pharmacy curricula and other learning experiences, and have created a foundation of PGx for this group, more so than any other health professional [144,145,146,147,148]. However, the substance of PGx content in curricula is inconsistent and still evolving as schools adjust to include PGx material in their curricula. The exact proportion of genetics materials regarding genomic technologies, variant interpretation, incomplete penetrance, and polygenic risks compared to pharmacological impact of genetic variation is not clear, and perhaps specialized tracks could be developed to align with students’ interests and schools’ expert faculties. Furthermore, continued efforts are needed to develop effective learning curricula and practice experiences to insure pharmacists possess the skills and knowledge to integrate PGx into practice [149,150,151]. For pharmacists in advanced training [152] as well as practicing pharmacists, a number of novel approaches have been developed, including experiential learning, online learning, clinical decision support and patient educational materials, and networks of PGx experts [78,153,154,155,156,157]. In particular, several schools and professional organizations have developed participatory or experiential learning opportunities for PGx testing [158,159,160,161]. Several schools and organizations now offer a Master’s degree or certificate programs in pharmacogenomics [162,163,164,165,166]. These programs may be especially attractive to pharmacists interested in establishing PGx testing as a major component of their clinical program or taking a leadership role to develop a testing program. With the anticipated growth of DTC PGx testing and consumers’ need for information about the value of these tests when considering purchasing and result interpretation after testing, particularly with respect to their medications, pharmacists are likely to be a logical consumer resource. Thus, aside from training offered by DTC companies or company sources, with an expanded educational foundation in PGx, pharmacists will be better able to assess the validity of tests and advise consumers appropriately.

Lastly, the inter-dependent relationship between pharmacists and prescribers may benefit from inter-professional educational programs in PGx [167,168].

## 5. Looking Ahead

While it is difficult to predict what the types of PGx testing and the specific mode(s) of delivery of testing will look like in 5–10 years, there is no reason to believe that the momentum of the field of PGx will not continue. Therefore, ongoing workforce preparation and supportive operations like CDS are needed to enable more widespread delivery of testing to patients in different clinical settings and by different providers. In a broader context, however, advances in sequencing technology, reduced costs [169], and greater public and patient awareness may increase utilization and access to more comprehensive testing platforms, including sequencing, which may be ordered for different clinical indications, but nonetheless will yield PGx information [170,171] and supersede targeted PGx testing. Regardless of the testing type or access to PGx test results, the need for pharmacists to fulfill one or more roles to facilitate the accurate interpretation and application of results to patients’ medications will not fade; rather, it will likely increase in necessity.

Overall, the plethora of studies thus far evaluating pharmacists in the delivery of PGx testing have been favorable, and support expanded roles in various hospital, clinical, and community settings. Pharmacist-initiated PGx testing could benefit both patients and prescribers alike by greatly improving patient access to testing, with pharmacists serving as experts and educational resources for both parties. Among the needs to be addressed to expand the role of pharmacists are continued efforts to prepare the next generation of pharmacists (and all prescribers) and to update practicing pharmacists on new developments. Although the pharmacy community has been very proactive in preparing pharmacists to deliver PGx testing, as the evidence basis continues to grow and evolve, more continuing education and clinical tools are needed to equip pharmacists with up-to-date knowledge and skills. For schools that lack access to faculty instructors trained in PGx, it would be extremely valuable to share general educational resources and online resources, and convene workshops at professional meetings for continued education that will close some educational gaps. New databases and CDS tools are absolutely necessary to ensure the accurate interpretation and application of results to medication decisions. In addition to didactic training in PGx, pharmacists will need training and experience with these tools in order to learn how to utilize them appropriately.

As more PGx testing programs are developed (likely by pharmacists) alongside the addition of PGx testing to pharmacy services, the roles of pharmacists may evolve as testing becomes more ubiquitous. Furthermore, more clinical guidelines may become available for other commonly used medications such as anti-diabetics, which will likely result in their increased utility in community settings. It is possible that delivery of PGx testing will shift toward more clinic or hospital-based settings rather than community settings, impacted by healthcare needs and reimbursement. The pharmacist interface between prescriber and patient presents many opportunities to offer counseling, consultation, and education to both parties. It is equally important to more clearly define the respective roles in the delivery of PGx testing between prescribers and pharmacists, as this may foster a more collaborative and trusting relationship, potentially resulting in higher acceptance rates of pharmacist recommendations. The inconsistency between pharmacist–provider educational requirements and opportunities regarding PGx has presented a major challenge to its clinical implementation, and has likely contributed lower acceptance rates in some instances; more general resources for all providers prescribing medications are needed to optimize the appropriate use of PGx testing, and the development of materials specifically for pharmacists to use or share with prescribers when discussing PGx testing may be greatly beneficial. The collaboration of pharmacists and prescribers to optimize the delivery and appropriate use of PGx is a key component in the successful use of PGx testing in different clinical settings. While many studies have been conducted at academic medical centers, community pharmacies offer the opportunity to expand testing to rural and other areas not associated with or accessible to an academic medical center. Lastly, after the challenging period of the pandemic and its impact on pharmacy services, the role of pharmacists in the delivery of PGx testing remains strong, and pharmacists may be in a better position to expand and include PGx testing as a core service. 

As prescribers become more familiar with testing, the roles of pharmacists may shift primarily toward patient education. With the expected arrival of DTC-PGx testing, which enables consumers/patients to order testing without a health provider and receive results directly, the pharmacist will play an important role in helping patients to consider the benefits and risks of testing, and to understand their test results post-testing. In addition, artificial intelligence is under development for genomics, including PGx [172]. Thus, the role of pharmacists and their educational needs will continue to evolve as new technologies and applications are developed.

## Figures and Tables

**Table 1:** The different roles of pharmacists; the scope of the role(s) may vary depending on the clinical setting, training, and pharmacist position.

Position	Roles
Administrative/Leadership	Conduct needs assessments; consult with clinical heads/administrators Identify a clinical testing laboratory or develop in-house PGx testing Lead development of clinical workflow for delivery of PGx testing Create/review/select operational support platforms (e.g., in-person pharmacist consultation services, clinical decision support systems) Negotiate payment/reimbursement Establish training programs (e.g., pharmacist residency)
Clinical Practice I (Pre-Testing)	Identify eligible patients for PGx testing based on prescription/medication history Perform patient education/counseling and obtain informed consent Consult with provider (provider education) and obtain provider authorization for testing Conduct medication therapy management Sample collection (e.g., buccal swabs)
Clinical Practice II (Post-Testing)	Perform patient counseling/communication of results Consult with providers and discuss clinical recommendations Conduct medication therapy management and incorporate PGx test results Review PGx results for new prescriptions

## Data Availability

Not applicable.
